# Identification of a high‐risk subtype of intestinal‐type Japanese gastric cancer by quantitative measurement of the luminal tumor proportion

**DOI:** 10.1002/cam4.1744

**Published:** 2018-08-29

**Authors:** Toru Aoyama, Gordon Hutchins, Tomio Arai, Kentaro Sakamaki, Yohei Miyagi, Akira Tsuburaya, Takashi Ogata, Takashi Oshima, Sophie Earle, Takaki Yoshikawa, Heike I. Grabsch

**Affiliations:** ^1^ Department of Gastrointestinal Surgery Kanagawa Cancer Center Yokohama Japan; ^2^ Section of Pathology and Tumour Biology Leeds Institute of Cancer and Pathology University of Leeds Leeds UK; ^3^ Department of Pathology Tokyo Metropolitan Geriatric Hospital Tokyo Japan; ^4^ Department of Biostatistics and Bioinformatics The University of Tokyo Tokyo Japan; ^5^ Molecular Pathology and Genetics Division Kanagawa Cancer Center Research Institute Yokohama Japan; ^6^ Department of Surgery Tsuboi Cancer Center Hospital Koriyama Japan; ^7^ Department of Gastric Surgery National Cancer Center Hospital Tokyo Japan; ^8^ Department of Pathology GROW ‐ School for Oncology and Developmental Biology Maastricht University Medical Center+ Maastricht The Netherlands

**Keywords:** gastric cancer, intestinal‐type, tumor proportion

## Abstract

**Background:**

We hypothesized that the relative proportion of tumor (PoT) at the luminal surface can predict gastric cancer (GC) patient survival.

**Methods:**

We measured the luminal PoT in resection specimens from 231 GC patients with stage II/III disease who had surgery at the Kanagawa Cancer Center, Yokohama, Japan. Tissue microarrays were used to assess the extent of immune cell infiltration by CD45 immunohistochemistry. Results were related to histopathological features and patient overall survival (OS).

**Results:**

PoT was significantly lower in diffuse‐type (30%) compared to intestinal‐type GC (41%), *P* = 0.03. Patients with low PoT intestinal‐type GC survived significantly longer than patients with high PoT intestinal‐type GC (5 years OS: 78% vs 47%, *P* = 0.0112). Low PoT was an independent favorable prognostic factor in multivariate analysis in intestinal‐type GC. Low PoT was correlated with high content of CD45‐positive immune cells (*P* = 0.035). There was no relationship between PoT and survival in diffuse‐type GC.

**Conclusions:**

This is the first study to identify a subgroup of patients with stage II/III intestinal‐type GC at high risk of recurrence by measuring PoT at the luminal surface. The relationship between PoT and immune cell content provides an initial insight into potential underlying biological mechanisms.

## 1 INTRODUCTION

Gastric cancer (GC) is the fourth most common malignant disease and the second most frequent cause of cancer‐related deaths worldwide.^1^ Complete resection is essential for the cure of localized gastric cancer.^2^ Patients with stage I disease, in which the tumor is limited to T1N0‐1 and T2N0, rarely develop a recurrence and have an excellent prognosis. Stage IV cancers are unresectable, and these patients have a very poor prognosis. On the other hand, GC patients with stage II/III disease often have recurrent disease even after complete curative resection.^3^ At present, the standard treatment for locally advanced GC in Asia, Europe, and the United States is D2 gastrectomy followed by adjuvant chemotherapy, surgery combined with pre‐ and postoperative chemotherapy, or surgery followed by postoperative chemoradiotherapy.^4^, ^5^, ^6^, ^7^

In recent years, the tumor microenvironment has become a focus of intense research, as alterations that occur in the stroma surrounding tumor cells or interactions between stroma and tumor cells might be related to patient prognosis and could represent potential new therapeutic targets.^8^, ^9^ The tumor microenvironment contains cells (fibroblasts, inflammatory cells, endothelial cells forming vascular channels, mesenchymal stem cells) and noncellular components such as extracellular matrix, deposited growth factors, and signaling molecules.^10^ Studies in selected cancer subtypes have quantified the components of primary tumors and suggested that the tumor composition is associated with patient survival.^11^, ^12^, ^13^ Our own previous genomic meta‐analysis identified a stromal gene expression signature as predictor of survival in gastric cancer.^14^ At the same time, we could demonstrate using quantitative morphometry that a high intra‐tumoral stroma content is related to poor survival in Caucasian GC patients which has been confirmed very recently by a study in Italian GC.^15^ Two studies from Asian GC seem to suggest a similar relationship between stroma content and survival. Data from the relative stroma content at the deep invasive tumor edge in Chinese GC was used to propose a “new” staging system combining tumor‐stroma ratio and conventional TNM staging.^16^ The study in Korean GC restricted their evaluation of the tumor stroma content to areas with greatest proportion of stroma in a series of signet ring cancers reaching similar conclusions.^17^

In order to optimize patient management decisions, prognostic factors should ideally be measurable at the time of diagnosis, for example, in the diagnostic biopsy. The methodology we used in our own previous study or was used by the Italian, Chinese, and Korean investigators depends on the availability of a resected specimen to select the area of interest for stroma evaluation.^15^, ^16^, ^17^, ^18^ It is unclear whether a similar relationship between tumor stroma content and GC patient survival can be demonstrated when measurements are performed in material from the luminal surface of the tumor.

The aim of our study was to investigate whether the relative proportion of tumor (PoT) measured in an area of highest tumor density at the luminal surface of the tumor (eg, in an area typically targeted by endoscopic biopsies) can predict survival in Japanese patients with stage II/III gastric cancer and hence may serve as a potential biomarker to identify patients at high risk of recurrence already at the time of diagnosis.

## 2 MATERIALS AND METHODS

### 2.1 Patients

The patients were selected from the prospective database of the Department of Gastrointestinal Surgery, Kanagawa Cancer Center, Yokohama, Japan, according to the following inclusion criteria; (a) histologically proven gastric adenocarcinoma, (b) patients underwent potentially curative D2 gastrectomy as primary treatment between June 2002 and March 2010, and (c) pathologically confirmed stage II or III disease (TNM classification 7th ed and 14th edition of the general rules for gastric cancer published by the Japanese Gastric Cancer Association).^19^, ^20^ A total of 231 patients fulfilled the inclusion criteria and were included in the current study. One hundred and six (45.9%) patients were treated by surgery alone, 104 (45.0%) by surgery followed by S‐1. 21 (9.1%) patients included in our study were recruited into the SAMIT trial and therefore received Tegafur/Uracil (UFT) adjuvant treatment. As adjuvant UFT treatment is not the standard adjuvant treatment for GC in JAPAN and the potential benefit of adjuvant UFT was uncertain at the time of our study, we excluded patients who received UFT for the survival analyses. Patients were followed‐up in the out‐patient clinic. Blood tests and physical examination were performed at least every 2 weeks in patients on adjuvant treatment, and at least every 3 months after finishing S‐1 treatment for 5 years. Patients in the surgery‐only group underwent similar examinations at least every 3 months. Patients had a computer tomography of the thorax and abdomen every 6 months during the first 3 years after surgery and then annually until 5 years after surgery.

### 2.2 Surgical procedure and pathological diagnosis

All patients had a distal or total gastrectomy with lymph node (LN) dissection for gastric cancer. In principle, a D1 or a D1+ lymphadenectomy is indicated for cT1N0 tumors, and D2 is applied for cN+ or cT2‐T4 tumors regardless of the approach. Spleen‐preserving D2 total gastrectomy was permitted in this study.

The resected stomach and all harvested LNs were fixed with 10% buffered formalin for at least 48 hours. After standard histopathological processing of the tissue, sections were cut from each block and stained with hematoxylin and eosin (H&E).

### 2.3 Measurement of the relative proportion of tumor (PoT)

**Figure 1:**
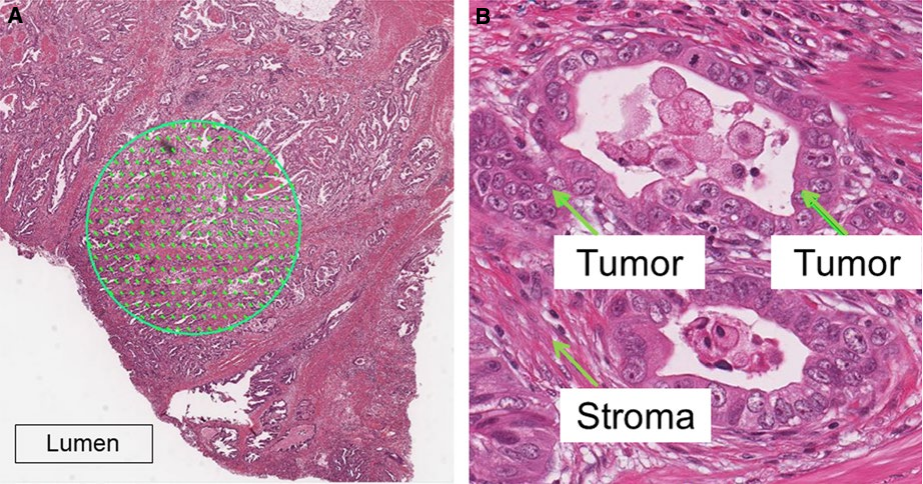
Morphometric method for establishing the proportion of tumor. A, Selection of a 9 mm^2^ area at the luminal surface of a hematoxylin and eosin‐stained representative section of gastric cancer. A total of 300 points are randomly inserted into the selected area. B, Annotation of three individual points comprised of tumor and stroma

Slides from all resection specimens were retrieved from the archive and reviewed by expert gastrointestinal pathologists. The H&E slide with the highest tumor cell density and the deepest tumor infiltration was selected. Slides were scanned at ×40 magnification with an automated scanning system (Aperio XT, Aperio Technologies, Vista, CA, USA) at the Slide Scanning Facility, LICAP, University of Leeds, UK. Using a digital slide viewer (Image Scope v8.0, Aperio Technologies), slides were reviewed and analyzed. A 9 mm^2^ area with highest tumor cell density on visual inspection was selected from the luminal surface as a surrogate for tissue sampling by endoscopic biopsies. A grid with a systematic random sample of 300 points was superimposed in the selected area using virtual graticule software (Random Spot, University of Leeds, Leeds, UK) to determine the relative proportion of the tumor components.^21^ Areas of necrosis and mucus at the surface were avoided when selecting the area (Figure 1). The following categories were used when evaluating the tissue at each measurement point: tumor, stroma, tumor lumen, necrosis, vessel, inflammation, and noninformative (unclassifiable). One of the authors (ToruA) was trained by experienced GI pathologists (HG & TA) in recognizing the different categories. All cases were scored by TA and ToruA blinded to the histopathological and survival data. To assess interobserver variation between experts, a random sample of 40 cases was also scored by a second GI pathologist (GH). PoT was expressed as a percentage fraction of tumor of the informative points per case. Each case took approximately 20 minutes to score.

### 2.4 Immunohistochemistry

After having obtained the results from the PoT measurement, we decided to expand our study and evaluate the overall extent of immune cell infiltration in the tumor hypothesizing that the extent of immune cell infiltration may provide some insights into the biology underlying our PoT results. Tissue microarrays (TMA) were constructed sampling two 1.5‐mm‐diameter cores from the same tumor area as described above as measurement area. Four micron sections were cut from each TMA block and manual immunohistochemistry (IHC) was carried out as described previously.^22^ Anti‐CD45 (clone 2B11/PD7/26 from Dako Cytomation) was used to stain all leucocytes and used as surrogate to assess the extent of immune cell infiltration in the tumor. A streptavidin biotin detection system (Dako REALTM Kit, Dako, Denmark) and 3,3′‐Diaminobenzidine (DAB) were used according to the manufacturer's instructions. Sections were counterstained with Mayer's Haemalaun, dehydrated and coverslipped. Sections from a multi‐tissue block served as positive and negative control.

### 2.5 Scoring of CD45 immunohistochemistry using automated image analysis

CD45 stained slides were scanned at x40 magnification using an Aperio XT digital slide scanner. The in‐house developed software system, Tissue MicroArray informatics (TMAi), was used to de‐array the digital images and assign the relevant identifiers to each core. Cores were individually analyzed using a custom‐made MATLAB‐based algorithm for the presence of foreground tissue stained brown with DAB based on the hue, saturation, and intensity (HSV) colour space. Detection thresholds were calculated per image as 0.5 standard deviation below the mean intensity of the staining channel. The percentage of positive pixels per core (Positive pixels/(positive pixels + negative pixels)*100) was used as a surrogate for the extent of immune cell infiltration per core. If results were available for more than one core from the same case, the mean value of the cores was calculated. Extensive manual quality control checks were carried out at every stage of the process. Thus, cores containing normal tissue only or a mixture of normal and tumor, with folds or other technical artifacts were excluded from the final analyses.

### 2.6 Statistical analyses

Comparisons between PoT, CD45 staining and clinicopathological variables were performed using the Mann‐Whitney U or Kruskal‐Wallis test as appropriate. Correlation analyses were performed using Spearman's rank correlation coefficients. Overall survival (OS) time was defined as the time from date of surgery to date of death or date of last follow‐up. The 21 patients who received adjuvant UFT was excluded from the survival analyses in order to have a homogenously treated study population. The relationship between OS and variable of interest was evaluated by uni‐ and multivariate analyses. OS curves were calculated using the Kaplan‐Meier method and compared by the log‐rank test. Cox's proportional hazard model was used to perform univariate and multivariate survival analyses. A *P*‐value of less than 0.05 was defined as statistically significant. The SPSS software package (v11.0J Win, SPSS, Chicago, IL, USA) was used for all statistical analyses.

This study was approved by the IRB Committee of the Kanagawa Cancer Center.

## 3 RESULTS

### 3.1 Clinicopathological data

**Figure 2:**
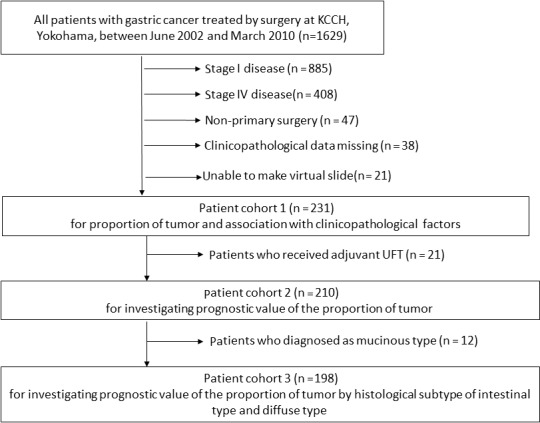
Consort diagram of the present study

The selection of the study population for the different analyses steps is illustrated in Figure 2. Median follow‐up time after surgery was 10.8 years (range: 6.1‐15.4 years). None of the patients died within 30 days after surgery. The median age was 65 years (range: 35‐85 years); 153 (66%) patients were male, and 78 were female. One hundred and twenty‐one patients had a distal gastrectomy, 110 a total gastrectomy. The clinicopathological data of the study population are summarized in Table 1.

**Table 1:** Relationship between clinicopathological data and proportion of tumor (whole series)

Characteristics	All cases		Proportion of tumor (cutoff (median): 33.55%)				*P* value
			Low (n = 115)		High (n = 116)		
	n	%	n	%	n	%	
Tumor location							
Upper third	68	29.4	39	33.9	29	25.0	0.205
Middle third	92	39.8	46	40.0	46	39.7	
Lower third	71	30.8	30	26.1	41	35.3	
Tumor size (mm)							
Median (range)	55 (15‐212)		55 (15‐200)		55 (18‐212)		0.437
Histological type							
Intestinal	78	33.8	30	26.1	48	41.4	**0.030**
Diffuse	141	61.0	80	69.6	61	52.6	
Mucinous	12	5.2	5	4.3	7	6.0	
Depth of invasion (pT)							
T2/T3	79	34.2	40	34.8	39	33.6	0.838
T4a	152	65.8	75	65.2	77	66.4	
Lymph node status (pN)							
N0	39	16.9	22	19.1	17	14.7	0.496
N1/N2/N3	192	83.1	93	80.1	99	85.3	
Lymphatic invasion							
Negative	83	36.0	40	34.8	43	37.1	0.717
Positive	148	64.0	75	65.2	73	62.9	
Venous invasion							
Negative	60	26.0	41	35.7	19	16.4	**0.001**
Positive	171	74.0	74	64.3	97	83.6	
Adjuvant chemotherapy							
Yes	125	54.1	63	54.8	62	53.4	0.839
No	106	45.9	52	45.2	54	46.6	

Significant p‐values in bold font.

### 3.2 Proportion of tumor (PoT)at the luminal surface

The median proportion of tumor (PoT) of the whole series was 33.55% (interquartile range from 0.31% to 88.6%). The relationship between clinicopathological variables and PoT (high vs low using the median as cutoff) is shown in Table 2. PoT was significantly lower in diffuse‐type GC compared to intestinal‐type GC. Venous invasion was more common in cancers with high PoT.

**Table 2:** Relationship between clinicopathological data and proportion of tumor by histological subtype

Characteristics	All cases	Proportion of tumor (intestinal‐type) (cutoff (median): 40.51%)					Proportion of tumor (diffuse‐type) (cutoff (median): 29.65%)				
		Low (n = 37)		High (n = 36)		*P*‐ value	Low (n = 62)		High (n = 63)		*P*‐value
	n (%)	n	%	n	%		n	%	n	%	
Tumor location											
Upper third	56 (28.3)	8	21.6	14	38.9	0.249	21	33.9	13	20.6	0.151
Middle third	83 (42.0)	14	37.8	12	33.3		28	45.1	29	46.0	
Lower third	59 (29.7)	15	40.6	10	27.8		13	21.0	21	33.3	
Tumor size (mm)											
Median (range)		50 (18‐95)		58 (20‐120)		0.082	54.5 (15‐200)		56.5 (20‐212)		0.474
Depth of invasion (pT)											
T2/T3	67 (33.8)	13	35.1	13	36.1	0.337	22	35.5	19	30.2	0.875
T4a	131 (66.2)	24	64.9	23	63.9		40	64.5	44	69.8	
Lymph node status (pN)											
N0	36 (18.2)	8	21.6	6	16.7	0.848	13	21.0	9	14.3	0.524
N1/N2/N3	162 (81.8)	29	78.4	30	83.3		49	79.0	54	85.7	
Lymphatic invasion											
Negative	74 (37.4)	14	37.8	14	38.9	0.926	24	38.7	22	34.9	0.661
Positive	124 (62.6)	23	62.2	22	61.1		38	61.3	41	65.1	
Venous invasion											
Negative	52 (26.3)	11	29.7	4	11.1	**0.049**	27	43.5	10	15.9	**0.001**
Positive	146 (73.7)	26	70.3	32	88.9		35	56.5	53	84.1	
Adjuvant chemotherapy											
Yes	98 (49.5)	13	35.1	21	58.3	**0.047**	33	53.2	31	49.2	0.653
No	100 (50.5)	24	64.9	15	41.7		29	46.8	32	50.8	

Significant p‐values in bold font.

### 3.3 Survival analyses

There was no significant relationship between PoT and overall survival in the whole patient cohort using the median PoT (33.55%) as cutoff. Five‐year OS rate was 63.5% in patients with high PoT tumors and 67.0% in patients with low PoT tumors (*P* = 0.582).

**Figure 3:**
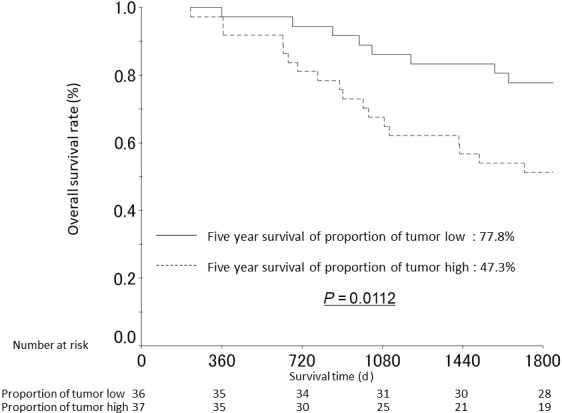
Overall survival curves from patients with intestinal‐type gastric cancer stratified by the proportion of tumor (low vs high based on median cutoff)

**Figure 4:**
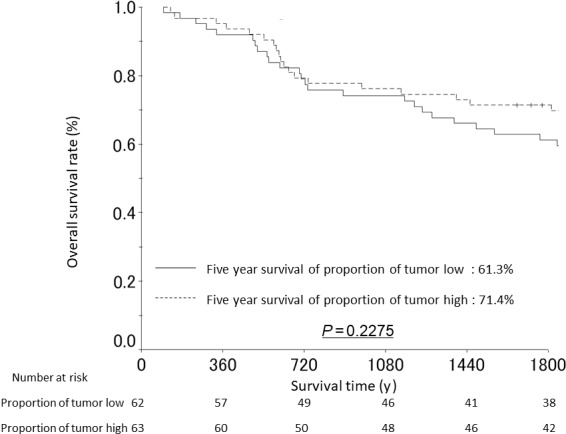
Overall survival curves from patients with diffuse type gastric cancer stratified by the proportion of tumor (low vs high based on median cutoff)

We noted that the median PoT was very different between intestinal‐type (40.51%) and diffuse‐type GC (29.65%) which prompted us to analyze the relationship with OS stratifying patients by histological tumor type. A significant relationship with OS was only seen in intestinal‐type GC using the intestinal‐type median PoT (40.51%) as cutoff for analyses. Patients with high PoT intestinal‐type GC had a significantly shorter 5‐year OS rate than patients with low PoT intestinal‐type GC (5‐year OS rate high PoT 47.3%, low PoT 77.8%; *P* = 0.0112) (Figure 3). Using Cox proportional hazards analysis, high PoT was associated with poorer OS in patients with intestinal‐type GC (hazard ratio (HR): 2.180, 95% confidence interval (CI): 1.087‐4.372, *P* = 0.028). Multivariate analysis confirmed that high PoT was an independent poor prognostic factor when the model was adjusted for age, pT, pN, and presence of venous invasion (*P* = 0.023, Table 3). Recurrences were more frequent in high PoT intestinal‐type GC. When analyzing PoT in diffuse‐type GC, we used the diffuse‐type median PoT (29.65%) as cutoff. The 5‐year OS rate was not significantly different between patients with high PoT diffuse‐type GC (71.4%) and patients with low PoT diffuse‐type GC (61.3%), *P* = 0.2275, Figure 4.

**Table 3:** Uni‐ and multivariate Cox proportional hazards analyses of the relationship between clinicopathological factors and overall survival in intestinal‐type gastric cancer

Characteristics	n (%)	Univariate analysis			Multivariate analysis		
		HR	95% CI	*P*‐value	HR	95% CI	*P*‐value
Age							
<65y	37 (50.7)	1.000		0.881			
≥65y	36 (49.3)	1.053	0.537‐2.065				
Site of tumor							
Lower or middle third	48 (65.8)	1.000		0.610			
Upper third	25 (34.2)	1.238	0.546‐2.808				
Tumor size							
<55mm	36 (49.3)	1.000		0.530			
≥55mm	37 (50.7)	1.242	0.631‐2.446				
Depth of invasion (pT)							
T2/T3	26 (35.6)	1.000		0.389	1.000		0.096
T4a	47 (64.4)	1.384	0.661‐2.896		1.762	0.904‐3.433	
Lymph node status (pN)							
N0	14 (19.2)	1.000		**0.005**	1.000		**0.001**
N1, N2, N3	59 (80.8)	2.157	1.264‐3.682		2.428	1.434‐4.111	
Lymphatic invasion							
Negative	28 (38.4)	1.000		0.829			
Positive	45 (61.6)	1.079	0.540‐2.157				
Venous invasion							
Negative	15 (20.5)	1.000		0.591			
Positive	58 (79.5)	1.273	0.527‐3.079				
Proportion of tumor							
Low	37 (50.7)	1.000		**0.028**	1.000		**0.023**
High	36 (49.3)	2.180	1.087‐4.372		2.271	1.122‐4.594	
Adjuvant chemotherapy							
Yes	39 (53.4)	1.000		0.567			
No	34 (46.6)	1.219	0.619‐2.399				

Significant p‐values in bold font.

**Figure 5:**
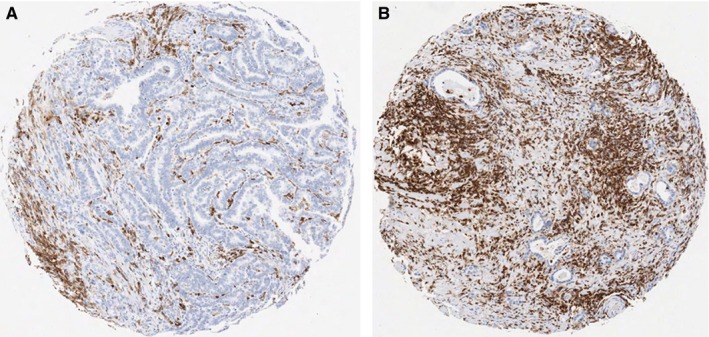
Relationship between proportion of tumor and extent of CD45‐positive immune cell infiltration in intestinal‐type gastric cancer. A, well differentiated intestinal type gastric cancer with low intratumoral stroma content shows relatively sparse infiltration by CD45‐positive immune cells. (CD45 positive cells = brown, counterstaining hematoxylin = blue). B, moderately differentiated intestinal‐type gastric cancer with high intratumoral stroma content and extensive infiltration by CD45‐positive immune cells. (CD45‐positive cells = brown, counterstaining hematoxylin = blue)

In order to explore potential underlying biological mechanisms of improved survival in patients with low PoT intestinal‐type GC, we quantified the overall immune response by CD45 immunohistochemistry in tissue microarrays using automated image analysis. Low PoT was correlated with high CD45 in intestinal‐type GC (Figure 5, *P* = 0.035).

## 4 DISCUSSION

In this study, we tested the hypothesis whether proportion of tumor (PoT) is related to survival in Japanese gastric cancer (GC) patients with locally advanced (TNM stage II/III) GC. In contrast to previous studies, we measured PoT at the luminal surface of the tumor as we wanted to evaluate whether PoT as a potential biomarker of high risk GC patients may be determined early on in patient management, for example, in diagnostic endoscopic biopsies.

As one might expect from routine histological GC typing, our study showed that the median PoT (eg, tumor cells per area tumor) was significantly higher in intestinal‐type GC which is usually characterized by glandular structures. The lower PoT in diffuse‐type GC might be related to the loss of adhesion between tumor cells in this tumor type which often infiltrates the tissue as single cells or small group of cells scattered in desmoplastic stroma.

To our surprise, our study showed that high PoT (and therefore low proportion of stroma) was a significant independent risk factor for poor overall survival in patients with intestinal‐type GC but not patients with diffuse‐type GC. Previous studies estimating stroma content in GC did not compare survival between patients with different histology type. We speculated that the immune cell infiltration, a parameter known to be related to GC patient survival, might be different in different histological subtypes and might depend on the extent of intratumoral stroma.^22^, ^23^ Our exploratory study using CD45 as pan‐leukocyte marker did confirm a correlation between stroma content, histological subtype, and CD45‐positive immune cells.

However, it is necessary to point out that the three previous GC studies evaluating the prognostic value of the proportion of stroma,^15^, ^16^, ^17^ but also studies investigating the same in colorectal cancer^18^ and other cancers^11^, ^12^, ^13^ all describe that a high proportion of stroma (eg, low PoT) is related to poor survival. Our study showed the opposite for the subgroup of intestinal‐type GC. The subgroup analysis of the diffuse‐type GC did not confirm the results from a previous study in signet ring cancers,^17^ although one has to realize that signet ring cancers are a specialized subgroup of diffuse‐type (or poorly cohesive type according to WHO) GC. Thus, a direct comparison between the results from signet ring cancers and diffuse‐type cancers does not appear to be feasible.

There are several differences between the current study and the previous studies which may explain the different results in our current study. We deliberately chose to measure the PoT at the luminal surface with the view to assess whether the prognostic (or predictive) value of PoT can potentially be measured in the diagnostic biopsy setting. All other studies measured PoT at the invasive edge, for example, an area which would not be accessible to biopsy sampling and/or focused on assessing areas with lowest tumor cell density whereas we focused on areas with highest tumor cell density at the opposite site of the tumor. Our study selected GC patients with stage II or stage III disease, whereas previous studies included patients with stage I, II, III, and IV. PoT might be related to disease stage which we could not reliably investigate in our study as patients were all either stage II or stage III disease. The study by Lee et al^17^ suggests that there could be a relationship between PoT and disease stage as in multivariate analysis the prognostic significance of the stroma proportion was only seen in GC patients with T3/4 disease. Furthermore, low PoT was seen in 8% of stage I patients and in up to 69% of stage III patients.^17^ While we measured PoT using a quantitative morphometric method, previous investigators simply visually estimated PoT which might explain the differences in the median PoT between studies.

Our study has some limitations. This study was a retrospective, single center study from Japan which might have lead to patient selection bias. Gastric cancer is known to be heterogeneous genetically and phenotypically. The measurement areas in the current study were selected in resection specimens based on a “presumed” endoscopic biopsy site. Unfortunately, we were unable to compare the results from the “virtual” endoscopic biopsies with that of the actual diagnostic biopsies of the same patients or with that of other parts of the tumor.

In conclusion, this is the first study to identify a subgroup of patients with intestinal‐type gastric cancer which has a very poor prognosis. The explorative analysis of the correlation between histological subtype, tumor content, and immune cell infiltration provides an initial insight into a potential underlying biological mechanism. Our study suggests that patient management decisions might not only need to consider Lauren's main histological subtypes (eg, intestinal or diffuse) as suggested recently, but also need to consider the tumor microenvironment (relative stroma content in combination with immune cell content) in particular in GC patients with intestinal‐type GC. The potential clinical impacts of our results warrant validation in a second independent GC cohort.

## CONFLICT OF INTEREST

The authors declare no conflicts of interest in association with the present study.

## HUMAN RIGHTS STATEMENT AND INFORMED CONSENT

The study data and informed consent were obtained in accordance with the Declaration of Helsinki and were approved by the Ethics Review Board of Kanagawa Cancer Center. Informed consent or substitute for it was obtained from all patients for　being included in the study.
